# Methylation pattern analysis in prostate cancer tissue: identification of biomarkers using an MS-MLPA approach

**DOI:** 10.1186/s12967-016-1014-6

**Published:** 2016-08-30

**Authors:** Giorgia Gurioli, Samanta Salvi, Filippo Martignano, Flavia Foca, Roberta Gunelli, Matteo Costantini, Giacomo Cicchetti, Ugo De Giorgi, Persio Dello Sbarba, Daniele Calistri, Valentina Casadio

**Affiliations:** 1 Biosciences Laboratory, Istituto Scientifico Romagnolo per lo Studio e la Cura dei Tumori (IRST) IRCCS, Via P. Maroncelli 40, 47014 Meldola, Italy; 2 Unit of Biostatistics and Clinical Trials, Istituto Scientifico Romagnolo per lo Studio e la Cura dei Tumori (IRST) IRCCS, Via P. Maroncelli 40, 47014 Meldola, Italy; 3 Department of Urology, Morgagni Pierantoni Hospital, Forlì, Italy; 4 Pathology Unit, Morgagni Pierantoni Hospital, Forlì, Italy; 5 Department of Urology, Bufalini Hospital, Cesena, Italy; 6 Department of Medical Oncology, Istituto Scientifico Romagnolo per lo Studio e la Cura dei Tumori (IRST) IRCCS, Via P. Maroncelli 40, 47014 Meldola, Italy; 7 Dipartimento di Scienze Biomediche, Sperimentali e Cliniche, University of Florence, Florence, Italy

**Keywords:** Methylation pattern, Prostate cancer, MS-MLPA, Early diagnosis

## Abstract

**Background:**

Epigenetic silencing mediated by CpG island methylation is a common feature of many cancers. Characterizing aberrant DNA methylation changes associated with prostate carcinogenesis could potentially identify a tumour-specific methylation pattern, facilitating the early diagnosis of prostate cancer. The objective of the study was to assess the methylation status of 40 tumour suppressor genes in prostate cancer and healthy prostatic tissues.

**Methods:**

We used methylation specific-multiplex ligation probe amplification (MS-MLPA) assay in two independent case series (training and validation set). The training set comprised samples of prostate cancer tissue (*n* = 40), healthy prostatic tissue adjacent to the tumor (*n* = 26), and healthy non prostatic tissue (*n* = 23), for a total of 89 DNA samples; the validation set was composed of 40 prostate cancer tissue samples and their adjacent healthy prostatic tissue, for a total of 80 DNA samples. Methylation specific-polymerase chain reaction (MSP) was used to confirm the results obtained in the validation set.

**Results:**

We identified five highly methylated genes in prostate cancer: *GSTP1, RARB, RASSF1, SCGB3A1, CCND2* (*P* < 0.0001), with an area under the ROC curve varying between 0.89 (95 % CI 0.82–0.97) and 0.95 (95 % CI 0.90–1.00). Diagnostic accuracy ranged from 80 % (95 % CI 70–88) to 90 % (95 % CI 81–96). Moreover, a concordance rate ranging from 83 % (95 % CI 72–90) to 89 % (95 % CI 80–95) was observed between MS-MLPA and MSP.

**Conclusions:**

Our preliminary results highlighted that hypermethylation of *GSTP1*, *RARB*, *RASSF1*, *SCGB3A1* and *CCND2* was highly tumour-specific in prostate cancer tissue.

**Electronic supplementary material:**

The online version of this article (doi:10.1186/s12967-016-1014-6) contains supplementary material, which is available to authorized users.

## Background

Prostate cancer (PCa) is the second most common malignancy in men [1]. Current diagnostic methods have increased the number of patients diagnosed with early PCa, with a consequent benefit in survival, but have also led to overtreatment, reducing the cost-effectiveness of treatment [2, 3]. Low sensitivity and specificity of the prostate-specific antigen (PSA) test, along with false-positive results and unnecessary biopsies for PSA values between 4 and 10 ng/ml, have created an urgent need for new biomarkers for PCa diagnosis [4, 5]. New biomarkers for PCa diagnosis have been studied with the purpose of improving sensitivity and specificity of PSA; some of these are FDA-approved, e.g. PCA3 testing in urine [6], but none has been implemented into clinical practice. For this reason, new, robust markers to accurately characterize PCa must be sought.

It is well known that epigenetic modifications such as DNA methylation in CpG islands are correlated with cancer development, indicating that such events could represent early phenomena of carcinogenesis [7]. For this reason, DNA methylation could be a potential biomarker for the early diagnosis of PCa. Tumour suppressor genes are sometimes silenced by methylation and contribute to carcinogenesis, influencing mechanisms such as DNA repair and apoptosis [8]. Moreover, every tumour type has a specific methylation pattern which, when compared with corresponding healthy tissue, could be useful for diagnosis [9]. Several studies have focused on the relationship between modifications of epigenetic mechanisms and prostate carcinogenesis [10].

In the present study we chose to analyze the methylation status of a panel of 40 tumour suppressor genes (*TIMP3, APC, CDKN2A, MLH1, ATM, RARB, CDKND2B, HIC1, CHFR, BRCA1, CASP8, CDKN1B, PTEN, BRCA2, CD44, RASSF1, DAPK1, VHL, ESR1, TP73, FHIT, CADM1, CDH13, GSTP1, CCND2, SCGB3A1, BNIP3, DLC1, HLTF, SFRP5, H2AFX, CACNA1AG, SFRP4, TWIST1, BCL2, CACNA1A, ID4, RUNX3, PRDM2* and *TGIF*) in PCa and healthy prostatic tissue to identify a tumour-specific methylation pattern that could facilitate early diagnosis. We chose this 40 genes panel because some of the selected genes (*GSTP1*, *TIMP3*, *RARB*, *CDKN2A*, *HIC*, *APC*, *CD44*, *RASSF1*, *CDH13*, *DAPK*, *BCL2*, *SFRP5*, *RUNX3*) are already known to be frequently methylated in PCa [11, 12]: *GSTP1* is the most widely studied and it is methylated in 70–80 % of cases [9]. In addiction other genes, e.g. *BRCA1*, *BRCA2*, *PTEN*, *TWIST1*, are deregulated in vitro and in vivo due to their low expression or other genomic characteristics [13, 14]. The remaining genes have important functions in different cancer-related processes, e.g. regulation of tumor growth, cell cycle control, differentiation and proliferation, cell adhesion and DNA damage repair.

We used the relatively new methodology of methylation specific-multiplex ligation dependent probe amplification (MS-MLPA) to evaluate epigenetic gene profiles in two independent cohorts of samples. This approach permits methylation analysis of multiple targets in a single experiment [15] and has been successfully used to evaluate the diagnostic or prognostic relevance of different markers in several tumor types including lung [16], rectal [17], breast [18] and bladder cancer [19, 20].

## Methods

### Case series

We analysed two independent case series: a training and validation set. For the training set, tissue samples were collected from patients submitted to prostatectomy between 2008 and 2011. Specifically, we collected 40 samples of paraffin-embedded PCa tissue, 26 of healthy prostatic tissue adjacent to the tumour, and 23 of healthy non prostatic tissue (seminal vesicles and bladder neck). In the training set we chose random samples, a number of which were not paired between cancer and healthy prostatic tissue. All paired cancer/healthy samples for the validation set were collected from patients who underwent prostatectomy in 2013. Specifically, we analysed 40 prostate cancer tissue samples and their adjacent healthy prostatic tissue, for a total of 80 samples.

All patients gave written informed consent to take part in the study, which was reviewed and approved by the local Ethics Committee (“Comitato Etico Area Vasta Romagna e IRST”). All samples were retrieved from the Archives of the Pathology Unit of the Morgagni-Pierantoni Hospital in Forlì.

### Macrodissection and DNA isolation

Cancer and healthy tissue was selected and macrodissected on the basis of hematoxylin-eosin sections. Healthy prostatic tissue was macrodissected at a distance of 7 mm from the tumour sample. DNA was extracted using QIAamp DNA FFPE Tissue (Qiagen, Milan, Italy), according to the manufacturer’s instructions, and quantified by spectrophotometry (NanoDrop ND-1000, Celbio, Milan, Italy). DNA from LNCaP cell line and peripheral blood of a healthy volunteer was extracted using QIAamp DNA Minikit (Qiagen), according to the manufacturer’s instructions.

### MS-MLPA

Methylation specific-multiplex ligation dependent probe amplification (MS-MLPA) was performed using at least 50 ng of DNA dissolved in 1× TE buffer (Promega, Madison, WI, USA). DNA isolated from LNCaP cell line was used as internal control for MS-MLPA analysis. The methylation status of 40 tumor suppressor gene promoters was analysed using the ME001-C1 and ME003-A1 kits (MRC-Holland, Amsterdam, The Netherlands) (Additional file 1: Table S1).

Two different probes that recognize two different sites of the promoter region were used for *RASSF1*, *MLH1*, *SCGB3A1*, *CCND2*, *ID4*, *RARB*, *SFRP4*, *DLC1*, *H2AFX* and *HLTF* genes. We considered the median value of the results of the two probes, assigning only one methylation value for each gene. *CDKN2B* gene was excluded from the analysis because its probe is sensitive to improper *Hha*I digestion in FFPE samples.

MS-MLPA analysis was performed following the manufacturer’s instructions. In brief, DNA was denatured and hybridization was performed by incubation at 60 °C for 16–18 h. Ligation and digestion reactions were then performed and samples were amplified by PCR. Digested probes could not be amplified exponentially during PCR and thus did not produce an amplification product. In contrast, if the DNA sample was methylated, DNA-probe hybrids were protected against *Hha*I enzyme digestion and the ligated probes generated an amplification product.

Amplification products were analysed by ABI-3130 genetic Analyzer (Applied Biosystem, Foster City, CA). Electropherograms obtained were evaluated using Gene Mapper software (Applied Biosystem) and the peak areas of each probe were exported to a homemade excel spreadsheet.

In accordance with the manufacturer’s instructions, we carried out “intrasample data normalization” by dividing the signal of each probe by the signal of each reference probe in the sample, thus creating as many ratios per probe as there were reference probes. We then calculated the median value of all probe ratios per probe, obtaining the normalization constant (NC). Finally, the methylation status of each probe was calculated by dividing the NC of a probe in the digested sample by the NC of the same probe in the undigested sample, and by multiplying this ratio by 100 to have a percentage value, as follows:

$$\frac{NC\;digested\;sample}{NC\;undigested\;sample} \times 100$$

We performed MS-MLPA analysis on four samples of peripheral blood of healthy volunteers, as controls for our analysis, finding absence of hypermethylation for all genes.

MS-MLPA reproducibility was assessed by performing three independent methylation profile analyses on LNCaP cell line. The methylation level for each gene was found to be the same in each experiment.

### MSP

We used MSP as a confirmatory methodology to analyse the promoter methylation of five genes: *GSTP1, RARB, RASSF1A, SCGB3A1* and *CCND2*. DNA was converted with sodium bisulphite using EZ DNA Methylation-Gold™ kit (Zymo Research Corporation, Irvine, USA). The reactions were performed using 100 ng of DNA extracted from LNCaP (methylated control) and from the peripheral blood of a healthy volunteer (unmethylated control), and 150 ng of FFPE DNA.

We performed real-time PCR using SYBR-GREEN master mix (Biorad, Milan, Italy) and primers specific for bisulphite-converted. Primer sequences for *Actin B* were as follows: forward 5′-TGGTGATGGAGGAGGTTTAGTAAGT-3′, reverse 5′-AACCAATAAAACCTACTCCTCCCTTAA-3′, as described elsewhere [21]. Real-time PCR was performed using Rotor Gene 3000 (Diatech pharmacogenetics, Jesi, Italy) under the following conditions: 95 °C for 5 min, then 40 cycles of 94 °C for 30 s, 62 °C for 60 s and 72 °C for 60 s. We then evaluated PCR product specificity with melt curve analysis and set the Ct threshold at 0.02. For the subsequent preamplification PCR, 10 µl of converted DNA was used when the Ct average value was ≥28, or 6 µl when <28.

We performed a two-step MSP for *RARB*, *RASSF1A*, *SCGB3A1* and *CCND2* methylation analysis, as previously described by Zhu et al. [22]. First, we performed a multiplex PCR using C1000™ Thermal Cycler (Biorad) containing 4 primer pairs (called ‘outer’) for each gene with AmpliTaq Gold PCR kit (Applied Biosystems) [22]. The outer primers targeted methylated and unmethylated sequences in the same loci to enhance the amount of the four specific fragments used as templates for the second real-time PCR. Primer sequences for *RARB*, *RASSF1A, SCGB3A1* and *CCND2* are described in Table 1. Real-time PCR was carried out using SYBR-GREEN master mix (Biorad) and specific primers for methylated and unmethylated sequences (Table 1) for each gene. The previous preamplification products were diluted 1 to 10,000 for *RARB*, *RASSF1A* and *SCGB3A1* and 1–5000 for *CCND2.* The reaction was performed under the following conditions: 95 °C for 5 min, then 40 cycles of 94 °C for 30 s, 58 °C for 60 s, and 72 °C for 60 s.

**Table 1:** Primer sequences

Gene	Outer primer sequences	Methylated primer sequences	Unmethylated primer sequences
*GSTP1*		5′-TATCGTGGTTTATTTTTTAGTTCGA-3′ 3′-ATAAAAAAATTCGAATCTCTCCGA-5′	5′-TATTGTGGTTTATTTTTTAGTTTGA-3′ 3′-ATAAAAAAATTCAAATCTCTCCAAA-5′
*RARB*	5′-TATGCGAGTTGTTTGAGGATTGGGA-3′ 3′-AATAATCATTTACCATTTTCCAAACTTA-5′	5′-TGTGAGAACGCGAGCGATTC-3′ 3′-CGACCAATCCAACCGAAACGA-5′	5′-TTGGGATGTTGAGAATGTGAGTGATTT-3′ 3′-CTTACTCAACCAATCCAACCAAAACAA-5′
*RASSF1*	5′-GTTTAGTTTGGATTTTGGGGGAG-3′ 3′-CCCACAACTCAATAAACTCAATAAACTCAAACTC-5′	5′-GGGTTCGTTTTGTGGTTTCGTTC-3′ 3′-TAACCCGATTAAACCCGTACTTCG-5′	5′-GGGGTTTGTTTTGTGGTTTTGTTT-3′ 3′-AACATAACCCAATTAAACCCATACTTCA-5′
*SCGB3A1*	5′-AGTGAGGATATTTAGAGAAATTTAGG-3′ 3′-ATCCCTACCTCTAATCCCAA-5′	5′-GCGTCGAGGTTAGTTCG-3′ 3′-GTAAACGCCTTCTACGCCTAA-5′	5′-GTGTTGAGGTTAGTTTTGAAGA-3′ 3′-TAAACACCTTCTACACCTAAAA-5′
*CCND2*	5′-TATTTTTTGTAAAGATAGTTTTGATTTAAGG-3′ 3′-TTTCCCCGAAAACATAAAACCTCC-5′	5′-GGCGGATTTTATCGTAGTCG-3′ 3′-CTCCACGCTCGATCCTTCG-5′	5′-AGAGTATGTGTTAGGGTTGATT-3′ 3′-ACATCCTCACCAACCTCCA-5′

For *GSTP1* we only performed real-time PCR on 2 µl of bisulphite-converted DNA samples. Primers for methylated and unmethylated sequences are shown in Table 1. The reaction was performed under the following conditions: 95 °C for 3 min, 40 cycles of 94 °C for 30 s, 56 °C for 60 s and 72 °C for 60 s.

### Statistical analysis

The sample size was calculated on the basis of the Bittner formula [23]: assuming α = 0.01, β = 0.10, a standard deviation of gene methylation intensity of measurements on the base-two logarithmic scale = 0.7 and a 1.5-fold difference between the two classes (normal and tumour tissue). We compared clinical-pathological features in the training and validation sets using a non parametric statistical test. A two-dimensional unsupervised hierarchical cluster analysis of the methylation profile was performed using Euclidean distance as similarity measure, and clusters were combined using Ward’s method. Normality of data distribution was tested using the Shapiro–Wilk test. If data distribution was not normal (*P* > 0.05), a non-parametric statistical test was used.

The relationship between methylation value and different subgroups of patients was analysed using the non-parametric statistical test (Wilcoxon test). For PCa samples, the relationship between methylation value and clinical-pathological features of patients were analysed using a non-parametric statistical test (Spearman correlation for PSA levels considered as continuous variable and Wilcoxon for the other variables).

In validation set, the genes showing a significant *P* value in the Wilcoxon test were used to analyse the most discriminant cut-off values between PCa and P using ROC curve analysis. The true positive rates (sensitivity) were plotted against the false positive rates (1-specificity) for all classification points. Cohen’s kappa coefficient was calculated to measure the concordance rate of MS-MLPA and MSP methods. A kappa value of more than 0.60 was regarded as showing strong agreement.

A stepwise logistic regression model was used in the validation set to analyse the relative risks (RR) and their 95 % CIs for patient status (PCa/P) and methylation status for selected genes.

All *P* values reported were two-sided and evaluated at the 0.05 level. Correction for multiple testing was performed using the Benjamini–Hochberg approach. Cluster analysis was performed with R software (version 3.0.1). All other statistical analyses were performed with STATA/MP 10.1 for Windows (Stata Corp LP).

## Results

**Fig. 1:**
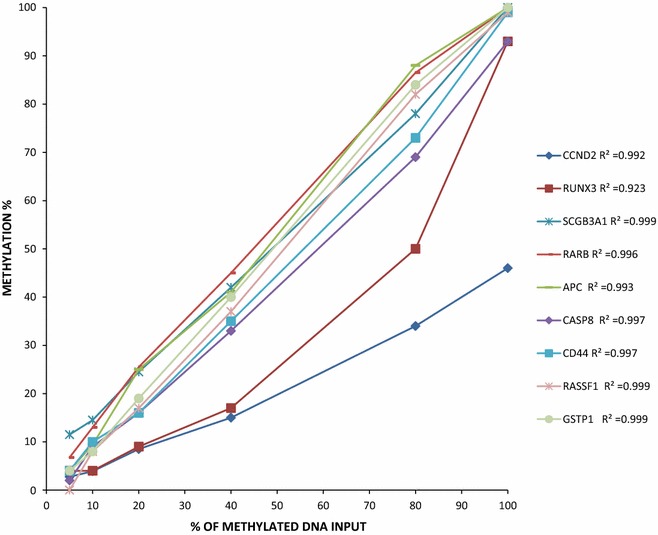
Correlation between different percentages of methylated DNA input (LNCaP cell line, *X axis*) and methylation percentage results obtained using the MS-MLPA technique (*Y axis*). Results for *CCND2, RUNX3, SCGB3A1, RARB, APC, CASP8, CD44, RASSF1* and *GSTP1* are reported with corresponding R^2^ results

The clinical-pathological characteristics are summarized in Table 2. We analysed two independent sets of samples: a training and a validation set. The sensitivity of the MS-MLPA technique was evaluated by constructing a curve based on the generation of different proportions (5, 10, 20, 40, 80, 100 %) of DNA derived from a prostate cancer cell line (LNCaP) with known methylation of *GSTP1, RASSF1, SCGB3A1, CASP8, RARB, CD44, APC, RUNX3, CCND2*, spiked in genomic DNA (control) from a blood sample of a healthy donor. We found good linearity and a positive correlation between methylated DNA input and MS-MLPA results (Fig. 1). We also showed that the MS-MLPA technique is capable of detecting very low methylation percentages (at least 5 %).

**Table 2:** Case series

	Training set *n* = *40*	Validation set *n* = *40*	*P* value^a^
Age, years			
≤70	32	33	0.775
>70	8	7	
Gleason score			
≤6	18	13	0.251
>6	22	27	
Pathological stage			
T2a	5	5	
T2b	1	0	
T2c	17	16	0.554
T3a	12	17	
T3b	5	2	
Median PSA (range)	6.77 (3.19–33.14)	5.81 (2.65–24.00)	0.1988

^a^The two groups were equally distributed for age, Gleason score, pathological stage and PSA. The Chi square test was used for age and Gleason score to determine statistical differences between training and validation sets; the Fisher test was used for pathological stage and the Wilcoxon test for PSA value

### Training set

**Fig. 2:**
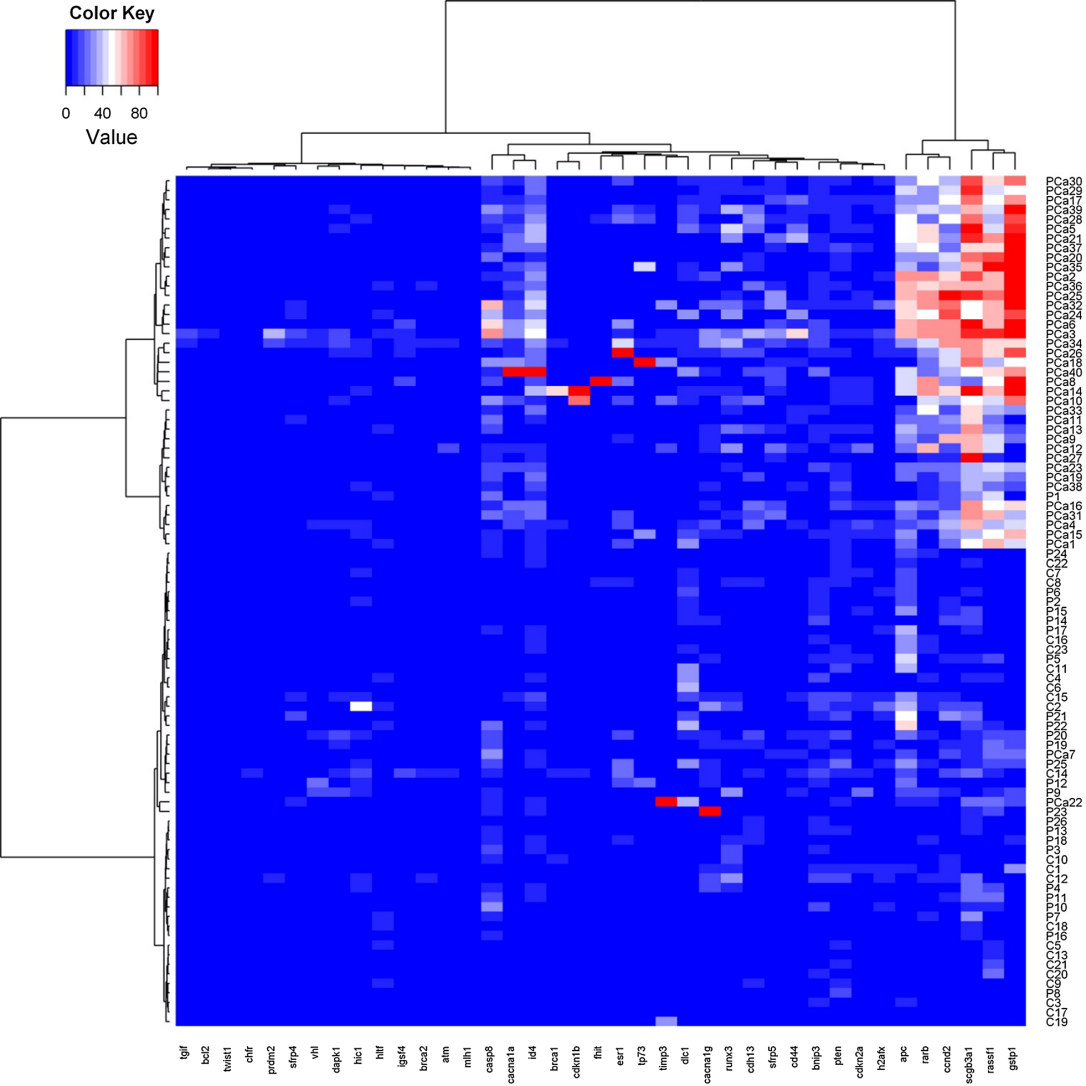
Hierarchical cluster analysis of methylation status of 40 tumour suppressor genes (training set): the *blue colour* indicates an absence of methylation in the genes, whereas *red indicates* high methylation; *shades of colour* indicate intermediate methylation status. The 40 genes are shown along the *bottom*, while the samples are represented in the column on the *right*

The methylation status of 40 tumour suppressor genes was analysed in 89 samples. Unsupervised cluster analysis for methylation profile identified two principal groups: one composed mainly of PCa tissue, the other mainly by healthy prostatic tissue adjacent to the tumour (defined as ‘P’) and healthy non prostatic tissue (‘C’) (Fig. 2). The median methylation levels for each gene in the three groups of samples are shown in Additional file 2: Table S2. Considering healthy and control samples, we observed statistically different methylation status for only 2 genes: the median methylation status of *CASP8* was 9.92 (range 0.00–28.90) in P vs. 0.00 (range 0.00–9.50) in C (*P* < 0.0001) and 9.45 (range 0.00–28.80) in P vs. 0.00 (range 0.00–20.80) in C for *SCGB3A1* (*P* = 0.0005) (Additional file 3: Figure S1). With the exception of these two genes, there were no differences in the methylation profile of the P and C samples. We compared the methylation status of the PCa and P groups to test the role of methylation in the early diagnosis of PCa. Comparative analysis showed that 12 genes had statistically higher methylation in tumour tissue (Table 3). No significant correlations were found between methylation status and clinical-pathological characteristics.

**Table 3:** Difference in methylated genes between cancer (PCa) samples and healthy (P) samples adjacent to the tumour

Gene	Training set			Validation set			AUC Validation set (95 % CI)
	Median value (range)		*P* value*	Median value (range)		*P* value*	
	PCa	P		PCa	P		
*GSTP1*	69.70 (0.00–100.00)	0.00 (0.00–18.20)	<0.0001	35.50 (0.00–100.00)	2.81 (0.00–30.18)	<0.0001	0.89 (0.82–0.97)
*RASSF1*	49.25 (11.10–100.00)	5.00 (0.00–45.60)	<0.0001	46.69 (9.88–79.98)	9.63 (0.00–74.81)	<0.0001	0.92 (0.85–0.98)
*CCND2*	35.10 (4.40–94.60)	5.75 (0.00–27.10)	<0.0001	33.29 (7.26–100.00)	5.41 (0.00–38.45)	<0.0001	0.92 (0.86–0.98)
*SCGB3A1*	65.90 (12.40–100.00)	9.45 (0.00–28.80)	<0.0001	56.47 (0.00–100.00)	10.35 (0.00–35.04)	<0.0001	0.95 (0.90–1.00)
*RARB*	29.95 (4.60–71.50)	4.65 (0.00–16.10)	<0.0001	35.89 (2.29–100.00)	4.55 (0.00–18.71)	<0.0001	0.94 (0.88–0.99)
*ID4*	22.65 (4.30–100.00)	5.15 (0.00–11.90)	<0.0001	14.41 (0.00–45.32)	5.08 (0.00–16.08)	<0.0001	0.86 (0.78–0.94)
*CACNA1A*	12.95 (0.00–100.00)	0.00 (0.00–2.13)	0.0001	6.32 (0.00–68.89)	0.00 (0.00–14.94)	0.0001	0.74 (0.64–0.84)
*SFRP5*	7.50 (0.00–29.30)	0.00 (0.00–0.00)	<0.0001	8.35 (0.00–39.01)	0.00 (0.00–10.79)	<0.0001	0.82 (0.73–0.91)
*APC*	32.55 (0.00–71.00)	4.88 (0.00–59.10)	0.0001	36.77 (0.00–91.80)	5.89 (0.00–38.90)	<0.0001	0.84 (0.75–0.93)
*CD44*	5.60 (0.00–57.40)	0.00 (0.00–10.30)	0.0009	5.92 (0.00–59.33)	4.08 (0.00–29.70)	0.1391	–
*CDH13*	0.00 (0.00–39.00)	0.00 (0.00–14.40)	0.0087	9.43 (0.00–41.21)	5.99 (0.00–27.62)	0.0557	–
*RUNX3*	3.75 (0.00–44.10)	0.00 (0.00–29.60)	0.0088	9.71 (0.00–47.22)	8.38 (0.00–44.90)	0.3351	–

*AUC* area under ROC curve

* Wilcoxon test: prostate cancer samples (PCa) vs. healthy adjacent prostate samples (P)

### Validation set

**Fig. 3:**
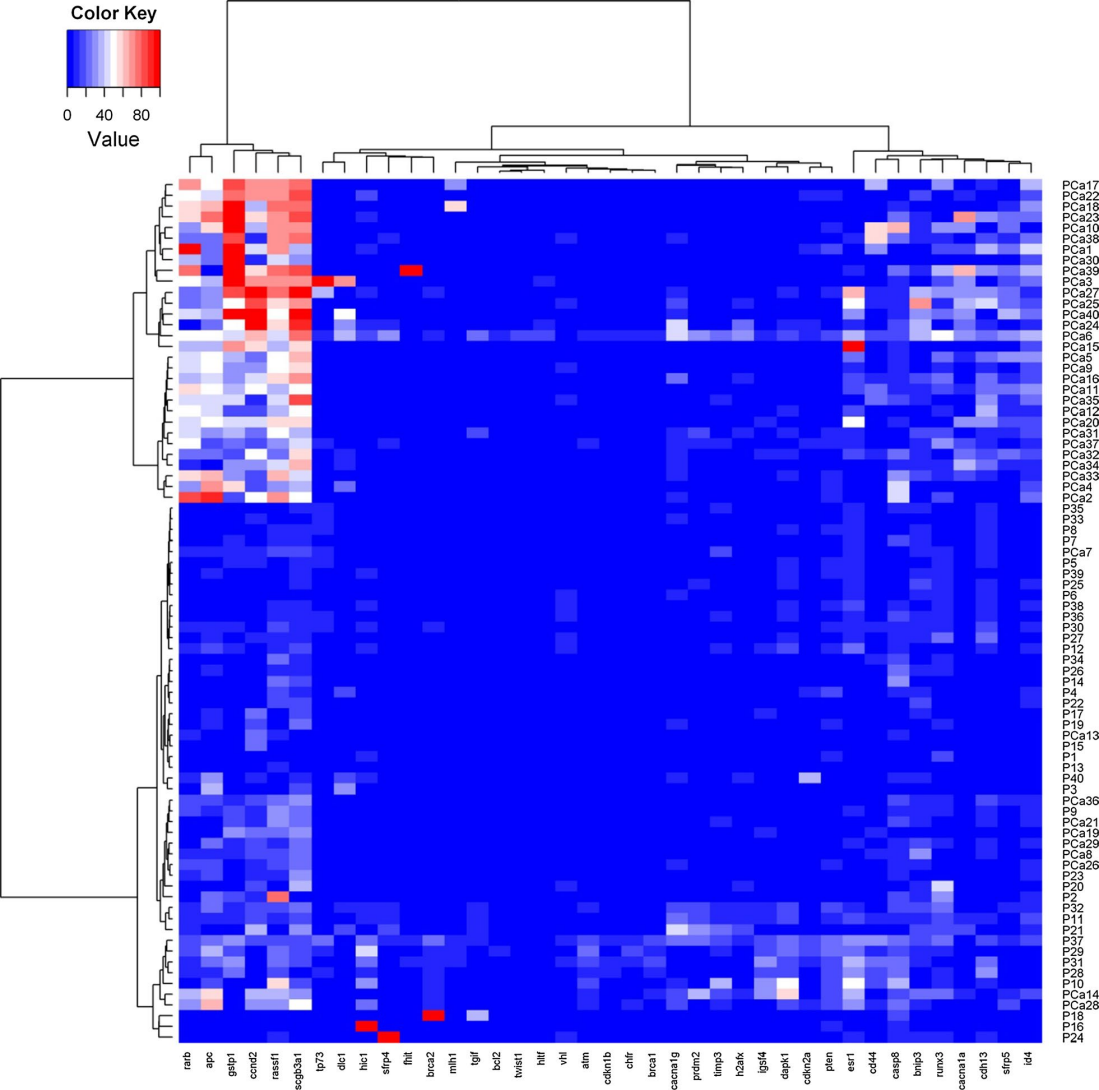
Hierarchical cluster analysis of methylation status of 40 tumour suppressor genes (validation set): the *blue colour* indicates an absence of methylation in the genes, whereas *red indicates* high methylation; *shades of colour* indicate intermediate methylation status. The 40 genes are shown along the *bottom*, while the samples are represented in the column on the *right*

**Fig. 4:**
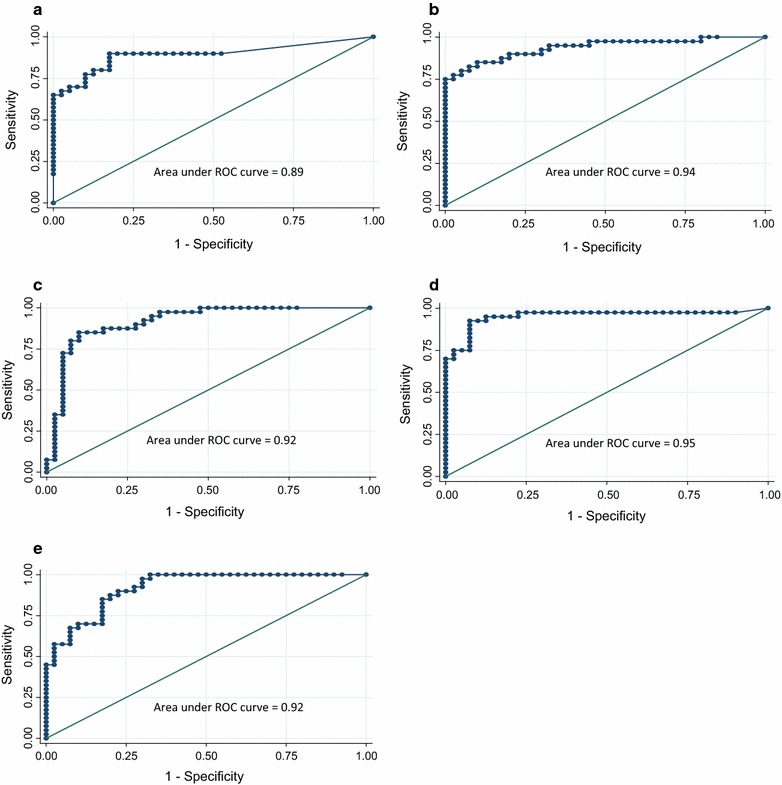
ROC curve analysis of the five genes highly specific in discriminating prostate cancer from healthy tissue: **a** *GSTP1*, **b** *RARB*, **c** *RASSF1*, **d** *SCGB3A1*, and **e** *CCND2*

Forty PCa tissue samples and 40 P samples were evaluated for the same panel of tumour suppressor genes. As in the training set, we identified two cluster groups in validation set, one composed of PCa samples, the other of P samples, the latter showing a lower methylation profile (Fig. 3). The median methylation levels for each gene in the two groups of samples are shown in Additional file 2: Table S2. Comparative analysis confirmed that gene methylation status differed between tumour and healthy tissue in 9 out of 12 genes (Table 3). We then performed receiver operating characteristic (ROC) curve analyses for each of these genes (Fig. 4): five genes (*GSTP1, RARB, RASSF1, SCGB3A1* and *CCND2*) were highly specific in discriminating between prostate cancer and adjacent normal tissue, with an area under ROC curve (AUC) ranging from 0.89 (95 % CI 0.82–0.97) to 0.95 (95 % CI 0.90–1.00). ROC curve analysis also identified the best methylation cut-off: we considered the promoters showing a ratio ≥0.20 as methylated, while those with a ratio <0.20 were considered as unmethylated. The overall diagnostic accuracy for the five genes varied between 80 and 90 % (Table 4). A stepwise regression analysis with a 0.20 probability removal was carried out in the validation set to evaluate the capability of *GSTP1, RARB, RASSF1, SCGB3A1 and CCND2* gene methylation to predict the risk of PCa. *RARB* and *SCGB3A1* proved to be independent variables that indicated a relative risk of there being prostate cancer of 1.14 (95 % CI 0.99–1.31, *P* = 0.058) and 1.10 (95 % CI 1.01–1.21, *P* = 0.028), respectively.

**Table 4:** Diagnostic accuracy

Gene	Overall sensitivity (n = 40) %	Early tumors sensitivity (n = 22) %^a^	Locally advanced tumors sensitivity (n = 18) %^b^	Overall specificity (n = 40) %	Overall accuracy (n = 80) %
*RARB*					
Rate (95 % CI)	30/40 75 (59–87)	15/22 68 (49–87)	15/18 83 (66–100)	40/40 100 (91–100)	70/80 88 (78–94)
*GSTP1*					
Rate (95 % CI)	28/40 70 (53–83)	14/22 64 (44–84)	14/18 78 (59–97)	37/40 93 (80–98)	65/80 81 (71–89)
*CCND2*					
Rate (95 % CI)	27/40 68 (51–81)	12/22 55 (34–76)	15/18 83 (66–100)	37/40 93 (80–98)	64/80 80 (70–88)
*RASSF1*					
Rate (95 % CI)	35/40 88 (73–96)	18/22 82 (66–98)	17/18 94 (83–100)	33/40 83 (67–93)	68/80 85 (75–92)
*SCGB3A1*					
Rate (95 % CI)	38/40 95 (83–99)	21/22 95 (86–100)	17/18 94 (83–100)	34/40 85 (70–94)	72/80 90 (81–96)

^a^Early tumors: GS ≤ 6 or T2

^b^Locally advanced tumors: GS > 6 or T3

Correlation analysis of methylation status and clinical-pathological characteristics showed that *RUNX3* was significantly correlated with PSA levels: its methylation status was positively associated with PSA levels (*P* = 0.0001). We also performed methylation specific PCR (MSP) analysis for *GSTP1, RARB, RASSF1, SCGB3A1* and *CCND2* to confirm their methylation status previously identified with the MS-MLPA analysis. We used Cohen’s kappa coefficient to assess the concordance rate between MS-MLPA and MSP analyses. The kappa coefficient was greater than 0.6 for each gene, indicating strong agreement that ranged from 83 to 89 %.

We also evaluated the global methylation status of 28 paired cancer and normal samples using ELISA assay (MethyFlash Methylated DNA Quantification Kit, Epigentek, NY, USA) and obtained a global methylation percentage. Nineteen (68 %) patients showed a 5-mC (5-methylcytosine) % decrease in cancer tissue compared to normal adjacent tissue (data not shown).

We compared early tumors of PCa (defined as Gleason score ≤6 or T2 pathological stage) with normal prostate tissue (P), but we also compared all PCa with P. We identified the same statistically significant differences in the two comparisons except for these genes: *CDH13*, *RUNX3* in training set and *HIC1* in validation set. For training set, *CDH13* and *RUNX3* had statistical significance in all PCa vs. P comparison but not in early tumors. For the validation set, *HIC1* acquired statistical significance in early tumors but this was not confirmed in all PCa series vs. P.

## Discussion

Aberrant DNA methylation usually occurs at an early stage in cancer, rendering DNA methylation biomarkers good candidates for early cancer detection. In the present study we used an MS-MLPA approach to identify a panel of tumour suppressor genes differentially methylated in prostate cancer with respect to healthy tissue. The MS-MLPA method is a highly sensitive method [17, 20, 24] that is capable of identifying several promoter regions using a small quantity of DNA [25, 26]. We detected 12 genes with high methylation levels in tumour tissue compared to healthy tissue in the training set, nine of which were subsequently confirmed in the validation set. Of these nine genes, five (*GSTP1*, *RARB*, *RASSF1*, *SCGB3A1* and *CCND2*) discriminated between tumour and healthy tissue with a diagnostic accuracy of ≥80 %. As the MS-MLPA technique is designed to assess the methylation status of single CpG dinucleotides, a negative finding at a single CpG dinucleotide is not sufficient to rule out methylation at a given island. For this reason we confirmed the methylation status of these five genes by MSP, a simpler and less expensive method, based on bisulphite DNA conversion.

The concordance rate between MS-MLPA (based on *Hha*I enzymatic cleavage of unmethylated sequences) and MSP (based on bisulphite conversion of unmethylated cytosines to uracils) revealed strong agreement between the two methods.

Although hypermethylation in the promoter regions of tumour suppressor genes is often observed in cancer, global DNA hypomethylation occurs in cancer tissue with respect to normal tissue [27, 28]. In line with these literature data, we observed a lower 5-mC % in cancer compared to normal adjacent tissue in our small cohort of 28 patients (validation set) (data not shown).

Numerous studies investigating *GSTP1*, *RARB* and *RASSF1* DNA promoter methylation have reported that hypermethylation in prostate tumour tissue may represent a biomarker of early cancer diagnosis, in accordance with our results [29, 30]. There are few studies in literature on the hypermethylation of *SCGB3A1* and *CCND2* in prostate tumour tissue. *SCGB3A1* is a growth-inhibitory cytokine which is downregulated in the majority of prostate, breast, lung, pancreatic and nasopharyngeal cancers due to DNA promoter hypermethylation [31]. *CCND2* is a cell cycle-regulatory gene whose altered expression makes it function as an oncogene or tumor suppressor gene [32]. Padar et al. [33] observed an association between *CCND2* inactivation and promoter region hypermethylation, reporting a positive correlation between methylation frequency and high Gleason score group suggestive of a prognostic role of the gene. Although *SCGB3A1* and *CCND2* methylation in prostate cancer has been reported, the possibility of using these markers for the early diagnosis of PCa has never been fully investigated.

We also highlighted a similar methylation profile for healthy prostatic tissue adjacent to the tumour and healthy non prostatic tissue, with the exception of *CASP8* and *SCGB3A1*, which showed a statistically higher methylation in the former.

The methylation level of *SCGB3A1* gradually increased from low in healthy non prostatic tissue, to intermediate in healthy prostatic samples, and to high in PCa tissue, suggesting a role in early tumourigenesis. For this reason, we hypothesised that *SCGB3A1* could be an important biomarker for cancer in non cancerous prostatic tissue. Noteworthy, *RASSF1* and *SCGB3A1* had similar sensitivity values both in early and in locally advanced tumors (Table 4), thus demonstrating they could be potentially involved in the very earliest phases of carcinogenesis. This is an important characteristic for early diagnostic biomarkers.

In recent years, DNA promoter methylation has also been acknowledged as a biomarker to support clinical decision-making for suspected PCa. An important clinical problem is to determine which patients with a suspicion of PCa and initial negative biopsy should be referred for a second biopsy [34]. As some studies have recently suggested that false-negative first biopsies occur in around 20–25 % of patients [35–37], it is essential to unmask the cases of PCa among the negative core biopsies, thus eliminating the need for a repeat biopsy. Two studies, the MATLOC study [35] and the more recent DOCUMENT study [36] have shown that three methylation markers (*GSTP1*, *APC* and *RASSF1*) are capable of identifying PCa among negative core biopsies.

The role of *GSTP1*, *RARB* and *RASSF1* DNA promoter methylation as a non invasive biomarker for the early diagnosis of PCa has been widely investigated in body fluids [38, 39] using the MSP technique. A number of authors have also suggested that urine cell-free DNA could represent a source of cost-effective and non invasive biomarkers [36, 40–42], e.g. specific promoter region methylation. To the best of our knowledge, *SCGB3A1* and *CCND2* have not been hypothesised as potential non invasive biomarkers for early diagnosis and it would thus be interesting to analyse them in urine or plasma cell-free DNA.

## Conclusions

Our preliminary results, obtained in a single-institution study with limited enrolment, showed that the hypermethylation of *GSTP1*, *RARB*, *RASSF1*, *SCGB3A1* and *CCND2* was highly tumour-specific in prostate cancer tissue. We verified the potential of these markers in two independent case series and using two different techniques. Larger prospective studies are now needed to investigate these genes in body fluids.

## Additional files

10.1186/s12967-016-1014-6 Function and localisation of tumor suppressor genes.
10.1186/s12967-016-1014-6 Median methylation values.
10.1186/s12967-016-1014-6 Methylation percentages of the two genes (*CASP8* and *SCGB3A1*) in P, C and PCa samples that were differentially methylated in C and P samples in the training set. The figure highlights a higher methylation percentage for PCa than for healthy samples, but also a higher methylation percentage for P than for C samples in both genes.
