# Supplementary material for: Methylation pattern analysis in prostate cancer tissue: identification of biomarkers using an MS-MLPA approach
Source: J Transl Med. 2016 Aug 30;14(1):249. doi: 10.1186/s12967-016-1014-6 (PMC5006561; doi:10.1186/s12967-016-1014-6)
Supplement: Supplementary file 1 — 10.1186/s12967-016-1014-6 Function and localisation of tumor suppressor genes. [file 12967_2016_1014_MOESM1_ESM.docx]

**Table S1. Function and localisation of tumor suppressor genes**

| **Gene** | **Function** | **Localisation** |
| --- | --- | --- |
| TIMP metallopeptidase inhibitor 3 (TIMP3) | Gene related to invasion and metastasis | 22q12.3 |
| Adenomatous polyposis coli (APC) | WNT antagonist | 5q22 |
| Cyclin-dependent kinase inhibitor 2A (CDKN2A) | Cell cycle control gene | 9p21 |
| MutL homolog 1, colon cancer, nonpolyposis type 2 (MLH1) | Gene involved in DNA repair | 3p22.1 |
| Ataxia telangiectasia mutated (ATM) | Cell cycle control gene | 11q23 |
| Retinoic acid receptor, beta (RARB) | Cell differentiation and proliferation | 3p24.2 |
| Cyclin-dependent kinase inhibitor 2B (CDKN2B) | Cell cycle control gene | 9p21 |
| Hypermethylated in Cancer 1(HIC1) | Tumor suppressor gene | 17p13.3 |
| Checkpoint with forkhead and ring finger domains (CHFR) | Tumor suppressor gene | 12q24.33 |
| Breast cancer 1, early onset (BRCA1) | Gene related to genomic stability maintenance | 17q21.31 |
| Caspase 8, apoptosis-related cysteine peptidase (CASP8) | Apoptosis related gene | 2q33.2 |
| Cyclin-dependent kinase inhibitor 1B (CDKN1B) | Cell cycle control gene | 12p13.2 |
| Phosphatase and tensin homolog (PTEN) | Cell cycle regulator gene | 10q23.3 |
| breast cancer 2, early onset (BRCA2) | Gene related to genomic stability maintenance | 13q12.3 |
| CD44 molecule (Indian blood group) (CD44) | Cell-cell interaction mediator | 11p12 |
| Ras association (RalGDS/AF-6) domain family member 1 (RASSF1) | Tumor suppressor gene | 3p21.3 |
| death-associated protein kinase 1 (DAPK1) | Apoptosis-related gene | 9q34.1 |
| Von Hippel-Lindau tumor suppressor (VHL) | Tumor suppressor gene | 3p25 |
| Estrogen receptor 1 (ESR1) | Gene related to cell differentiation and proliferation | 6q25.1 |
| Tumor protein p73 (TP73) | Gene involved in apoptotic response to DNA damage | 1p36.32 |
| Fragile histidine triad gene (FHIT) | Tumor suppressor gene | 3p14.2 |
| Cell adhesion molecule 1 (IGSF4 (CADM1)) | Cell adesion-related gene | 11q23 |
| Cadherin 13, H-cadherin (heart) (CDH13) | Gene related to cell invasion | 16q23.3 |
| Glutathione S-transferase pi 1 (GSTP1) | Gene involved in DNA damage repair | 11q13 |
| Cyclin D 2 (CCND2) | Cell cycle control gene | 12p.13.3 |
| Secretoglobin, family 3A, member (SCGB3A1) | Tumor suppressor gene | 5q35 |
| BCL2/adenovirus E1B 19kDa interacting protein (BNIP3) | Apoptosis-related gene | 10q26.3 |
| Deleted in liver cancer 1 (DLC1) | Tumor suppressor gene | 8p22 |
| Helicase-like transcription factor (HLTF) | Gene related to genomic stability maintenance | 3q25.1 |
| Secreted frizzled-related protein (SFRP5) | Gene involved in cell differentiation and proliferation | 10q24.1 |
| H2A histone family, member (H2AFX) | Gene related to genomic stability maintenance | 11q23.3 |
| Calcium channel, voltage-dependent, T type, alpha 1G subunit (CACNA1G) | Gene involved in cell growth | 17q21.33 |
| Secreted frizzled-related protein (SFRP4) | Gene involved in cell differentiation and proliferation | 7P14.1 |
| Twist homolog 1 (TWIST1) | Gene involved in transcription regulation | 7p21.2 |
| B-cell CLL/lymphoma (BCL2) | Apoptosis-related gene | 18q21.3 |
| Calcium channel, voltage-dependent, P/Q type, alpha 1A subunit (CACNA1A) | Gene related to expression, cell motility, cell division and death | 19P13.2 |
| Inhibitor of DNA binding 4, dominant negative helix-loop-helix protein (ID4) | Tumor suppressor gene | 6p22.3 |
| Runt-related transcription factor 3 (RUNX3) | Tumor suppressor gene | 1p36.11 |
| PR domain containing 2, with ZNF domain (PRDM2) | Gene involved in transcription regulation | 1p36.21 |
| TGFB-induced factor homeobox (TGIF1) | Tumor suppressor gene | 18p11.31 |
