# Supplementary material for: Methylation pattern analysis in prostate cancer tissue: identification of biomarkers using an MS-MLPA approach
Source: J Transl Med. 2016 Aug 30;14(1):249. doi: 10.1186/s12967-016-1014-6 (PMC5006561; doi:10.1186/s12967-016-1014-6)
Supplement: Supplementary file 2 — 10.1186/s12967-016-1014-6 Median methylation values. [file 12967_2016_1014_MOESM2_ESM.docx]

**Table S2. Median methylation values**

| **Gene** | **Training set** | | | **Validation set** | |
| --- | --- | --- | --- | --- | --- |
|  | **PCa median (range)** | **P median (range)** | **C median (range)** | **PCa median (range)** | **P median (range)** |
| ***APC*** | 32.55 (0.00-71.00) | 4.88 (0.00-59.10) | 0.00 (0.00-36.50) | 36.77 (0.00-91.80) | 5.89 (0.00-38.90) |
| ***CDKND2A*** | 5.32 (0.00-23.37) | 0.00 (0.00-25.80) | 0.00 (0.00-10.30) | 2.62 (0.00-13.13) | 3.31 (0.00-37.00) |
| ***ATM*** | 2.55 (0.00-15.72) | 1.46 (0.00-4.70) | 0.00 (0.00-9.90) | 1.89 (0.00-12.67) | 2.41 (0.00-20.98) |
| ***HIC1*** | 0.00 (0.00-11.70) | 0.00 (0.00-9.50) | 0.00 (0.00-15.20) | 0.00 (0.00-21.29) | 3.12 (0.00-100.00) |
| ***CHFR*** | 0.00 (0.00-5.60) | 0.00 (0.00-2.20) | 0.00 (0.00-6.80) | 0.00 (0.00-8.11) | 0.00 (0.00-13.62) |
| ***BRCA1*** | 0.00 (0.00-58.70) | 0.00 (0.00-6.60) | 0.00 (0.00-9.60) | 0.00 (0.00-11.88) | 1.16 (0.00-15.54) |
| ***CASP8*** | 13.12 (0.00-72.40) | 9.92 (0.00-28.90) | 0.00 (0.00-9.50) | 8.91 (0.00-62.87) | 9.34 (0.00-33.42) |
| ***CDKN1B*** | 0.00 (0.00-100.00) | 0.00 (0.00-5.90) | 0.00 (0.00-9.10) | 2.36 (0.00-10.16) | 2.98 (0.00-12.89) |
| ***PTEN*** | 7.75 (0.00-17.10) | 5.05 (0.00-20.80) | 8.40 (0.00-17.70) | 5.60 (0.00-16.70) | 6.27 (0.00-25.47) |
| ***BRCA2*** | 0.00 (0.00-7.60) | 0.00 (0.00-6.20) | 0.00 (0.00-11.80) | 0.89 (0.00-12.84) | 2.35 (0.00-100.00) |
| ***CD44*** | 5.60 (0.00-57.40) | 0.00 (0.00-10.30) | 0.00 (0.00-10.50) | 5.92 (0.00-59.33) | 4.08 (0.00-29.70) |
| ***DAPK1*** | 0.00 (0.00-15.00) | 0.00 (0.00-15.70) | 0.00 (0.00-12.80) | 0.00 (0.00-54.14) | 2.85 (0.00-50.58) |
| ***VHL*** | 0.00 (0.00-12.00) | 0.00 (0.00-26.50) | 0.00 (0.00-5.90) | 0.00 (0.00-11.31) | 0.00 (0.00-11.93) |
| ***ESR1*** | 0.00 (0.00-100.00) | 0.00 (0.00-22.60) | 0.00 (0.00-24.80) | 6.83 (0.00-100.00) | 6.95 (0.00-52.03) |
| ***TP73*** | 0.00 (0.00-100.00) | 0.00 (0.00-21.20) | 0.00 (0.00-5.50) | 0.00 (0.00-100.00) | 0.00 (0.00-23.49) |
| ***FHIT*** | 0.00 (0.00-100.00) | 0.00 (0.00-5.50) | 0.00 (0.00-9.90) | 0.00 (0.00-100.00) | 0.00 (0.00-11.05) |
| ***IGSF4*** | 0.00 (0.00-19.40) | 0.00 (0.00-5.80) | 0.00 (0.00-14.40) | 0.00 (0.00-28.11) | 0.00 (0.00-28.32) |
| ***CDH13*** | 0.00 (0.00-39.00) | 0.00 (0.00-14.40) | 0.00 (0.00-13.30) | 9.43 (0.00-41.21) | 5.99 (0.00-27.62) |
| ***GSTP1*** | 69.70 (0.00-100.00) | 0.00 (0.00-18.20) | 0.00 (0.00-26.90) | 35.50 (0.00-100.00) | 2.81 (0.00-30.18) |
| ***BNIP3*** | 6.46 (0.00-25.30) | 6.80 (0.00-19.10) | 0.00 (0.00-17.00) | 10.14 (0.00-67.63) | 7.73 (0.00-19.52) |
| ***CACNA1G*** | 6.25 (0.00-32.30) | 0.00 (0.00-100.00) | 0.00 (0.00-32.40) | 5.20 (0.00-44.62) | 4.79 (0.00-41.51) |
| ***TWIST1*** | 0.00 (0.00-0.00) | 0.00 (0.00-4.00) | 0.00 (0.00-2.60) | 0.00 (0.00-16.74) | 0.00 (0.00-5.20) |
| ***BCL2*** | 0.00 (0.00-6.70) | 0.00 (0.00-0.00) | 0.00 (0.00-0.00) | 0.00 (0.00-10.70) | 0.00 (0.00-7.10) |
| ***CACNA1A*** | 12.95 (0.00-100.00) | 0.00 (0.00-2.13) | 0.00 (0.00-8.30) | 6.32 (0.00-68.89) | 0.00 (0.00-14.94) |
| ***RUNX3*** | 3.75 (0.00-44.10) | 0.00 (0.00-29.60) | 0.00 (0.00-29.40) | 9.71 (0.00-47.22) | 8.38 (0.00-44.90) |
| ***PRDM2*** | 0.00 (0.00-35.00) | 0.00 (0.00-3.99) | 0.00 (0.00-10.20) | 0.00 (0.00-33.54) | 0.00 (0.00-31.96) |
| ***TGIF*** | 0.00 (0.00-13.90) | 0.00 (0.00-2.26) | 0.00 (0.00-0.00) | 0.00 (0.00-24.02) | 0.00 (0.00-34.61) |
| ***TIMP3*** | 0.00 (0.00-100.00) | 0.00 (0.00-5.50) | 0.00 (0.00-32.20) | 1.42 (0.00-23.64) | 2.48 (0.00-37.47) |
| ***RASSF1*** | 49.25 (11.10-100.00) | 5.00 (0.00-45.60) | 0.00 (0.00-22.00) | 46.69 (9.88-79.98) | 9.63 (0.00-74.81) |
| ***DLC1*** | 3.85 (0.00-34.90) | 1.70 (0.00-33.50) | 0.00 (0.00-39.90) | 1.00 (0.00-71.32) | 0.92 (0.00-28.00) |
| ***CCND2*** | 35.10 (4.40-94.60) | 5.75 (0.00-27.10) | 2.00 (0.00-17.20) | 33.29 (7.26-100.00) | 5.41 (0.00-38.45) |
| ***H2AFX*** | 4.70 (0.00-20.60) | 4.20 (0.00-9.20) | 3.70 (0.00-23.30) | 3.30 (0.00-28.02) | 3.61 (0.00-18.56) |
| ***SCGB3A1*** | 65.90 (12.40-100.00) | 9.45 (0.00-28.80) | 0.00 (0.00-20.80) | 56.47 (0.00-100.00) | 10.35 (0.00-35.04) |
| ***HLTF*** | 2.30 (0.00-10.00) | 1.35 (0.00-9.70) | 2.20 (0.00-9.70) | 0.75 (0.00-18.12) | 0.17 (0.00-6.43) |
| ***ID4*** | 22.65 (4.30-100.00) | 5.15 (0.00-11.90) | 0.00 (0.00-16.70) | 14.41 (0.00-45.32) | 5.08 (0.00-16.08) |
| ***RARB*** | 29.95 (4.60-71.50) | 4.65 (0.00-16.10) | 4.20 (0.00-12.80) | 35.89 (2.29-100.00) | 4.55 (0.00-18.71) |
| ***SFRP4*** | 2.10 (0.00-13.48) | 0.00 (0.00-14.65) | 0.00 (0.00-8.78) | 2.49 (0.00-27.71) | 3.21 (0.00-100.00) |
| ***MLH1*** | 2.70 (0.00-4.50) | 2.35 (0.00-3.80) | 0.00 (0.00-5.70) | 1.86 (0.00-54.37) | 1.72 (0.00-9.61) |
| ***SFRP5*** | 7.50 (0.00-29.30) | 0.00 (0.00-0.00) | 0.00 (0.00-1.70) | 8.35 (0.00-39.01) | 0.00 (0.00-10.79) |
