# Supplementary figures and images for: Methylation pattern analysis in prostate cancer tissue: identification of biomarkers using an MS-MLPA approach
Source: J Transl Med. 2016 Aug 30;14(1):249. doi: 10.1186/s12967-016-1014-6 (PMC5006561; doi:10.1186/s12967-016-1014-6)

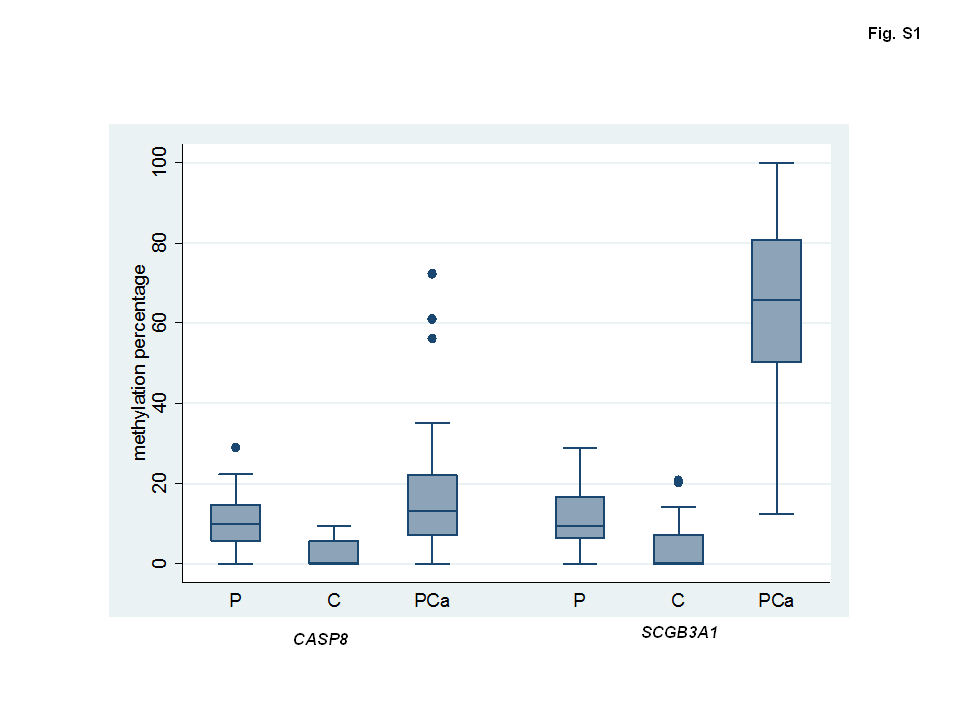

Supplement: Supplementary file 3 — 10.1186/s12967-016-1014-6 Methylation percentages of the two genes (CASP8 and SCGB3A1) in P, C and PCa samples that were differentially methylated in C and P samples in the training set. The figure highlights a higher methylation percentage for PCa than for healthy samples, but also a higher methylation percentage for P than for C samples in both genes. [file 12967_2016_1014_MOESM3_ESM.tif]
